# Author Correction: LOTUS domain protein MARF1 binds CCR4-NOT deadenylase complex to post-transcriptionally regulate gene expression in oocytes

**DOI:** 10.1038/s41467-019-09056-6

**Published:** 2019-03-01

**Authors:** Li Zhu, Suresh K. Kandasamy, Susan E. Liao, Ryuya Fukunaga

**Affiliations:** Department of Biological Chemistry, Johns Hopkins University School of Medicine, 725 North Wolfe Street, 521 A Physiology Building, Baltimore, MD 21205 USA

Correction to: *Nature Communications* 10.1038/s41467-018-06404-w, published online 02 October 2018

The originally published version of this Article contained an error in Fig. 1a, in which the length of the protein fragment produced by the MARF1 null allele was incorrectly labelled as ‘34aa’ rather than the corrected ‘103aa’. Also, the second sentence of the third paragraph of the Results originally read ‘The MARF1null allele has a 241-nt-long deletion introduced at proximal to the N-terminal end of the protein, which produced a premature stop codon, resulting in production of the N-terminal 34 aa fragment of MARF1 (Fig. 1a).’ In the corrected version, ‘34aa’ is replaced by ‘103aa’.These errors have now been corrected in both the PDF and the HTML versions of the Article.

