# Supplementary material for: LOTUS domain protein MARF1 binds CCR4-NOT deadenylase complex to post-transcriptionally regulate gene expression in oocytes
Source: Nat Commun. 2018 Oct 2;9:4031. doi: 10.1038/s41467-018-06404-w (PMC6168497; doi:10.1038/s41467-018-06404-w)
Supplement: Supplementary file 1 — Supplementary Information [file 41467_2018_6404_MOESM1_ESM.pdf]

Supplementary Information

LOTUS domain protein MARF1 binds CCR4-NOT deadenylase complex to post-transcriptionally regulate gene expression in oocytes

Zhu et.al.

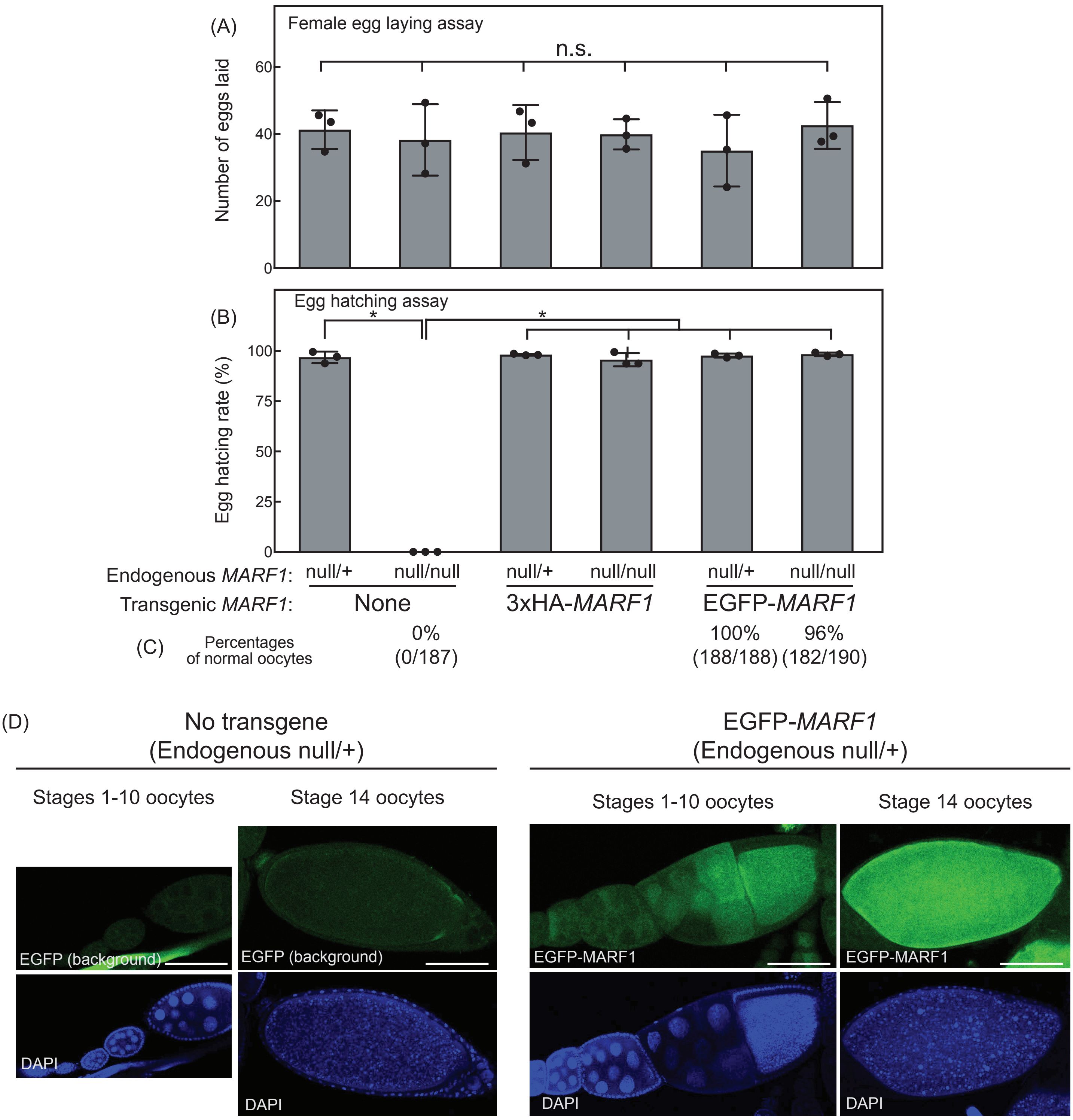

Supplementary Figure 1. EGFP-MARF1 does not exhibit particular localization in oocytes.
(A, B) Female fertility assays. (A) The numbers of eggs laid by test females crossed with OregonR wild-type males and (B) the hatching rates from the eggs. Mean +/- SD (n=3). P-value <0.05 (Student's t-test) are indicated by *.
(C) The percentages of normal stage 14 oocytes are shown. The numbers of normal oocytes/the numbers of observed oocytes are shown in parenthesis.
(D) Confocal images of the GFP signals in the oocytes without any transgene (left) and those expressing EGFP-MARF1 (right. MAT15-Gal4 -> UASP-EGFP-*MARF1*) in the endogenous *marf1* null/+ background.
Images of the oocytes without any transgene show the background GFP signals. EGFP-MARF1 was observed in both nucleus and cytoplasm without showing particular localization. Scar bars are 100 μm.

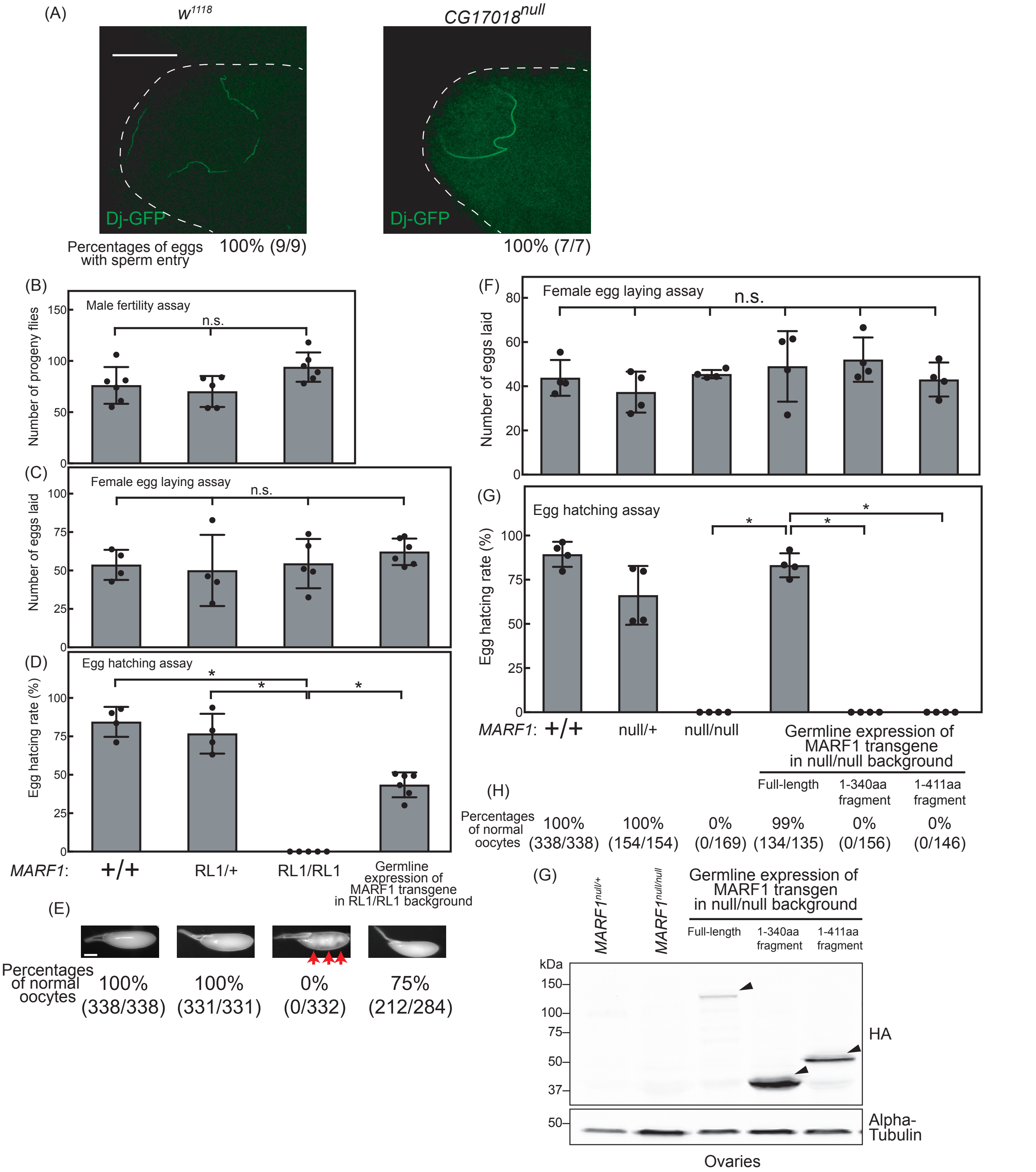

Supplementary Figure 2. Sperm can enter *MARF1*^{null} eggs properly and *MARF1*^{RL1} flies are female-sterile.
(A) Confocal images of the GFP signals of control (*w*^{1118}) and *MARF1*^{null} eggs mated with male flies expressing the sperm marker DJ-GFP. The green string signals indicate that sperm can enter properly into control and *MARF1*^{null} eggs. The numbers of eggs with the sperm GFP signal/the numbers of eggs observed are shown. Scar bar is 50 µm.
(B) Male fertility assay. The numbers of the progeny flies from crosses between test males and OregonR wild-type females are shown. Mean +/- SD (n=10).
(C, D) Female fertility assays. (C) The numbers of eggs laid by test females crossed with OregonR wild-type males and (D) the hatching rates from the eggs. Mean +/- SD (n=3). P-value <0.05 (Student's t-test) are indicated by *.
(E) Stereomicroscope images of stage 14 oocytes. The abnormal yolk distribution in *MARF1*^{RL1} stage 14 oocytes is indicated by red arrows. The percentages of normal oocytes are shown. The numbers of normal oocytes/the numbers of observed oocytes are shown in parenthesis. Scar bar is 100 µm. Germline-expression of the transgenic MARF1 protein (nanos-Gal4-VP16 -> UASP-3xHA-*MARF1*) in the *MARF1*$^{RL1/RL1}$ background rescued hatching rate.
(F, G) Female fertility assays. (F) The numbers of eggs laid by test females crossed with OregonR wild-type males and (G) the hatching rates from the eggs. Mean +/- SD (n=3). P-value <0.05 (Student's t-test) are indicated by *.
(H) The percentages of normal stage 14 oocytes are shown. The numbers of normal oocytes/the numbers of observed oocytes are shown in parenthesis. Germline-expression of the full-length transgenic MARF1 protein, but not the 1-340 aa or 1-411 aa fragment (MAT15Tub-Gal4 -> UASP-3xHA-*MARF1*) in the MARF1null/null background rescued hatching rate and stage 14 oocyte yolk distribution.
(I) Western blots of ovary lysates. In the anti-HA Western blot image, the transgenic 3xHA-MARF1 proteins are indicated by black triangles.

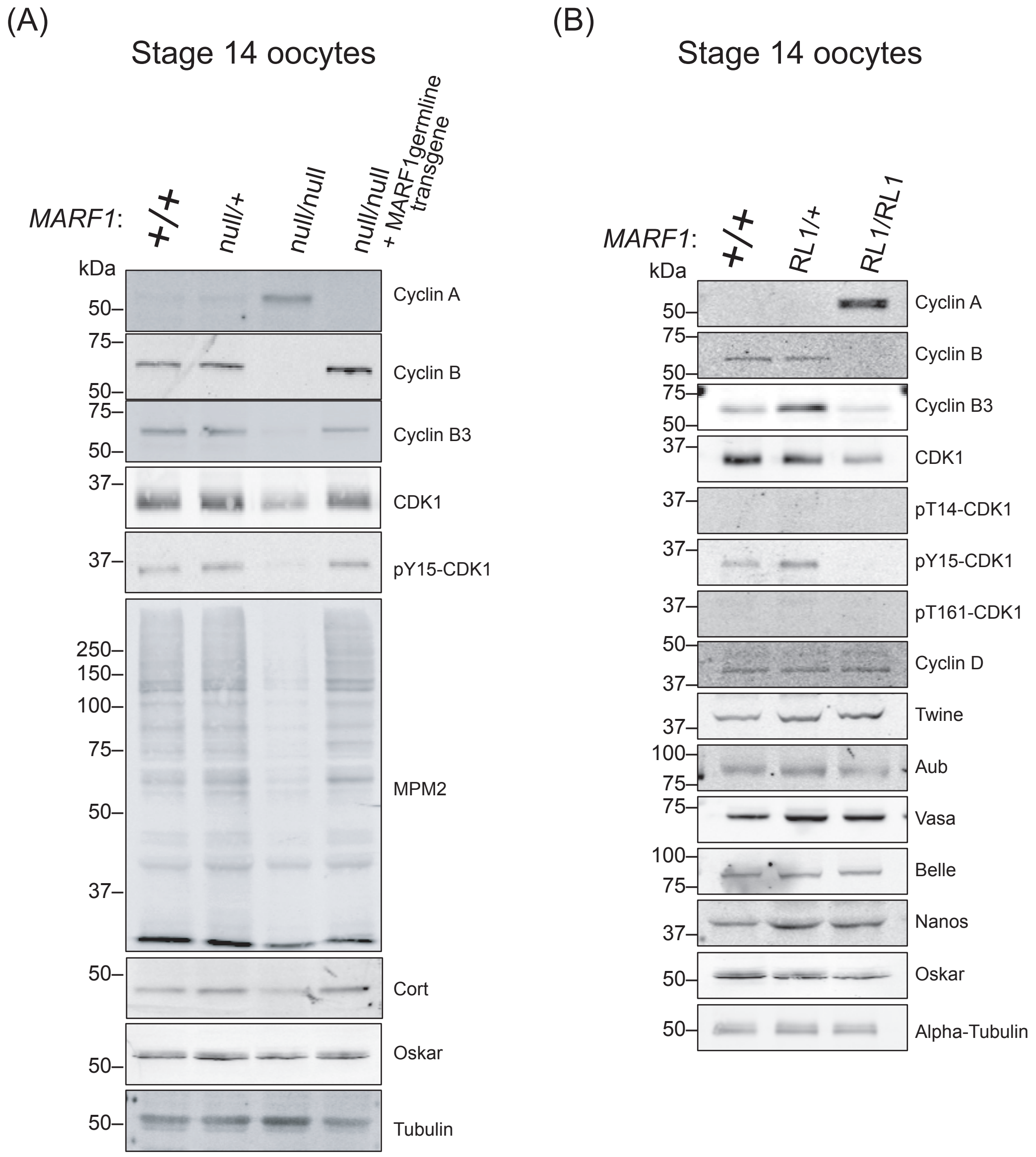

Supplementary Figure 3. Dysregulation of meiotic cyclin protein levels and protein phosphorylation pattern in *MARF1^{null}* and *MARF1^{RL1}* stage 14 oocytes.

(A) Western blots using control (*MARF1$^{+/+}$* and *MARF1$^{null/+}$*) and *MARF1$^{null/null}$* stage 14 oocytes. *MARF1$^{null/null}$* stage 14 oocytes rescued by germline expression of the transgenic MARF1 protein (MAT15Tub-Gal4 -> UASP-3xHA-*MARF1*) were also tested.

(B) Western blots using control (*MARF1$^{+/+}$* and *MARF1$^{RL1/+}$*) and *MARF1$^{RL1/RL1}$* stage 14 oocytes.

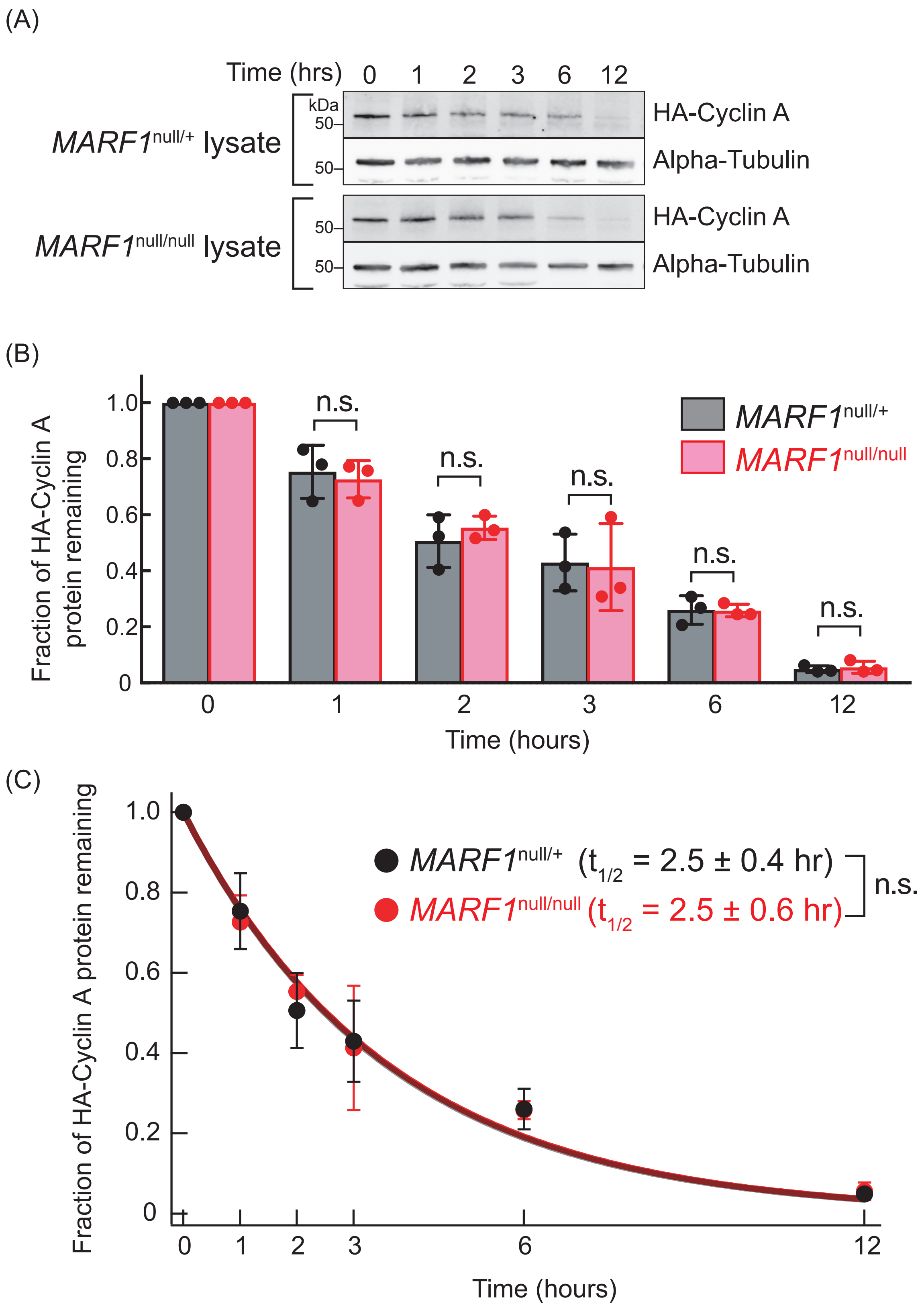

Supplementary Figure 4. Similar half-lives of recombinant Cyclin A protein between in *MARF1*$^{null/+}$ and *MARF1*$^{null/null}$ stage 14 oocyte lysates in vitro.
(A) In vitro degradation assay of recombinant HA-Cyclin A protein in *MARF1*$^{null/+}$ and *MARF1*$^{null/null}$ stage 14 oocyte lysates. Western blots to measure the levels of exogenously added recombinant HA-Cyclin A protein and endogenous alpha-Tubulin protein in stage 14 oocyte lysates in vitro during time course were shown.
(B) Quantification of the signals in (A). The fraction of the remaining recombinant HA-Cyclin A protein normalized by endogenous alpha-Tubulin protein was plotted. Mean +/- SD (n=3).
(C) Curve fitting to the data in (B).

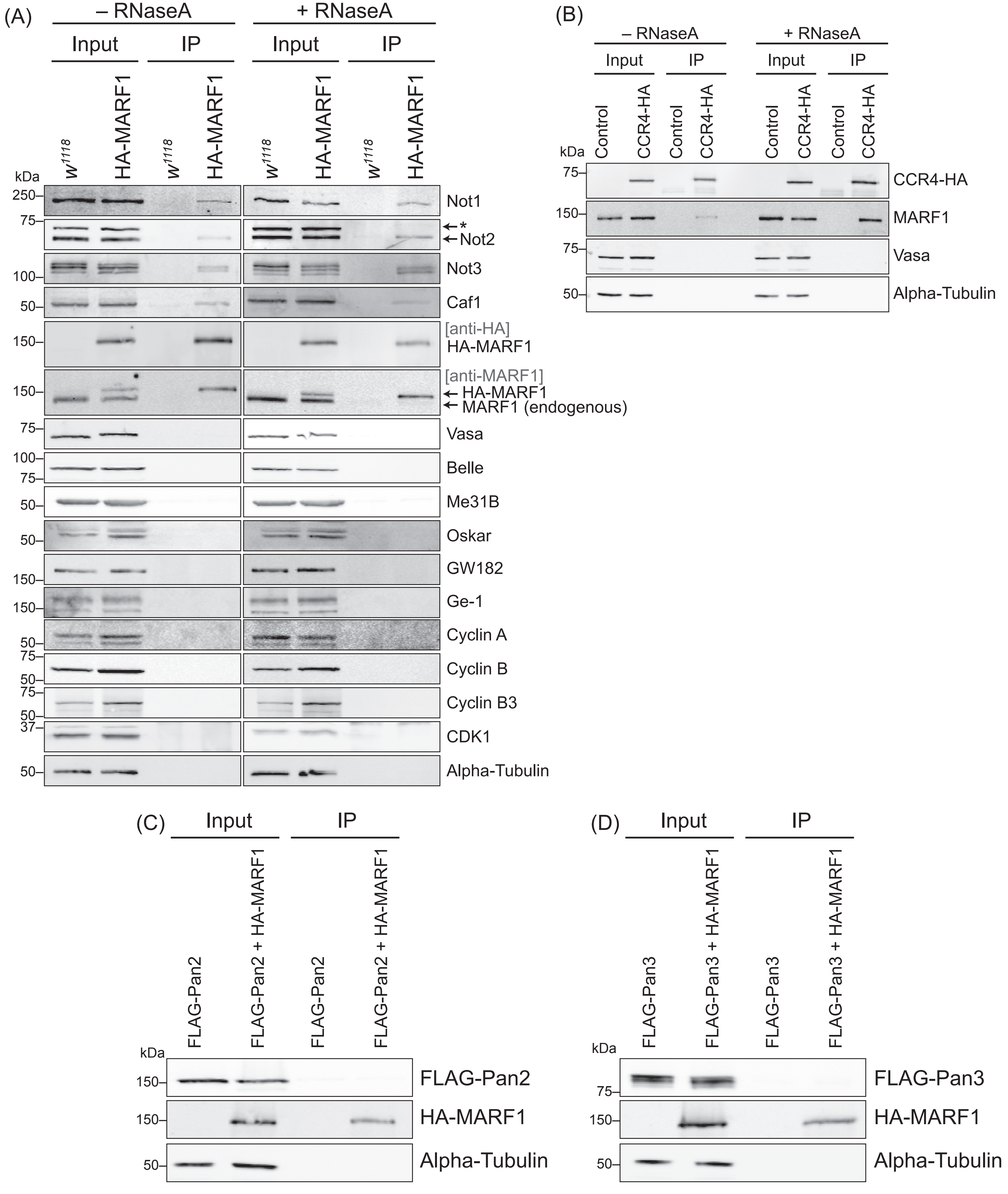

Supplementary Figure 5. MARF1 binds the CCR4-NOT deadenylase complex specifically.
Co-immunoprecipitation using anti-HA antibody-conjugated beads followed by Western blotting.
(A) Ovary lysates expressing 3xHA-MARF1 (MAT67Tub-Gal4 -> UASP-3xHA-*MARF1*) and those from w^{1118} negative control were tested.
(B) Ovary lysates expressing CCR4-HA (MAT67Tub-Gal4 -> UASP-*CCR4*-HA) and those from negative control (MAT67Tub-Gal4 only) were tested. Endogenous MARF1 was co-immunoprecipitated with CCR4-HA.
(C) Ovary lysates expressing 3xFLAG-Pan2 +/- 3xHA-MARF1 (MAT67Tub-Gal4 -> UASP-3xFLAG-*Pan2,* UASP-3xHA-*MARF1*) were tested.
(D) Ovary lysates expressing 3xFLAG-Pan3 +/- 3xHA-MARF1 (MAT67Tub-Gal4 -> UASP-3xFLAG-*Pan3,* UASP-3xHA-*MARF1*) were tested.

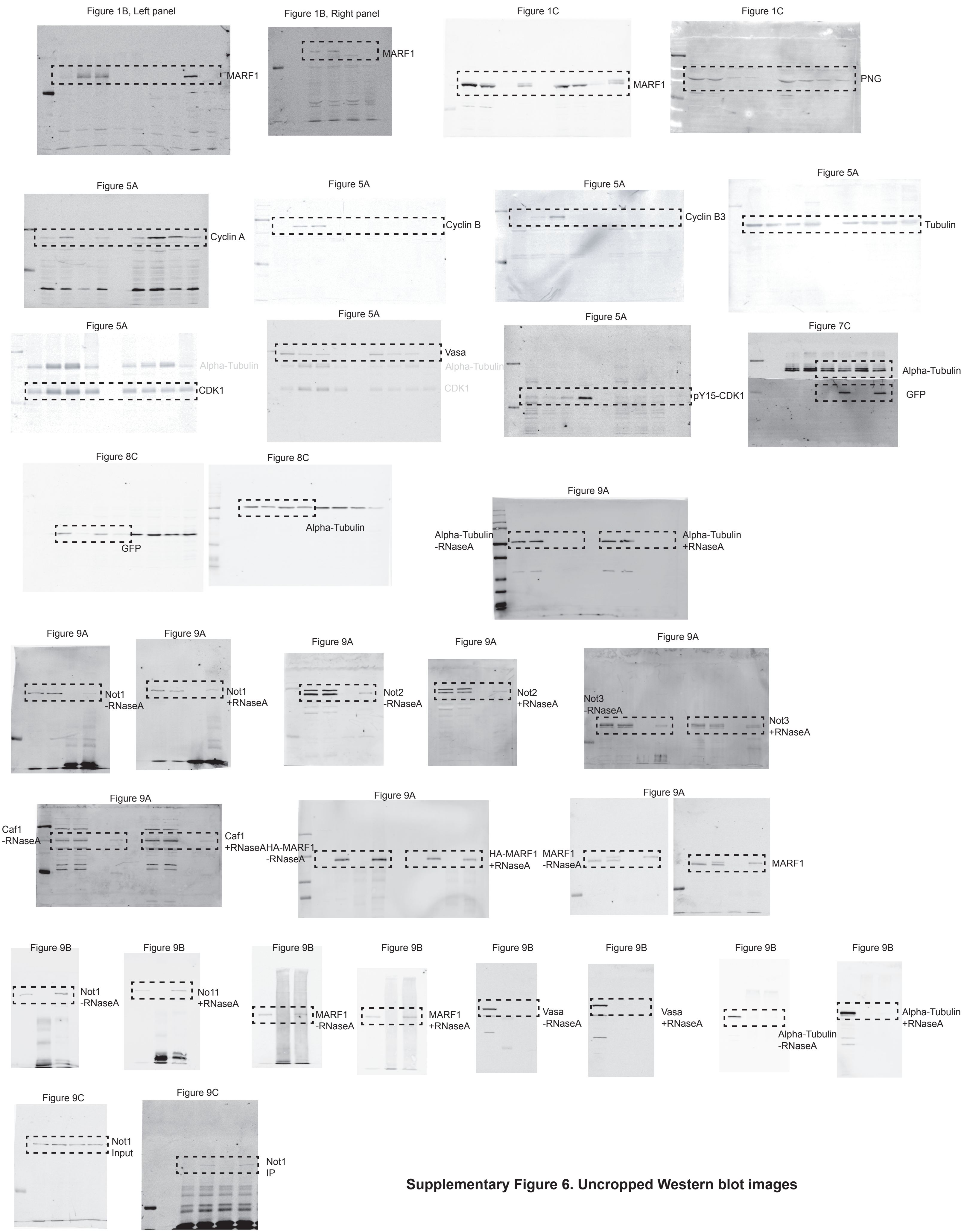

Supplementary Figure 6. Uncropped Western blot images

Supplementary Table 1.

List of oligonucleotide primers

ePAT assay	
Name	Sequence (5′ - 3′)
GFP-ePAT-F	GTCTCGATTCTACGCGTACCGG
cyclin A-ePAT-F	CAGCTAGCTTCGAGAATTATTTTACC
cdk1-ePAT-F	GATCCAGTTCATCGCATTTCCG
ePAT-R	GAGCTCCGCGGCCGCG
qRT-PCR	
GFP-F	GAGCTGAAGGGCATCGACTT
GFP-R	TTCTGCTTGTCGGCCATGAT
gapdh-F	TGATGAAATTAAGGCCAAGGTTCAGGA
gapdh-R	TCGTTGTCGTACCAAGAGATCAGCTTC
cyclin A-F	AAGAGTCAAGGAGCTTCCGC
cyclin A-R	TGTTTCTTCTCGCTCTCCCG
cyclin B-F	CCACTGTAGAACCCACTAAAGTTAC
cyclin B-R	GGTCAGCGACTTCTTCGACA
cyclin B3-F	ACCCTGGCTCGATACATCCT
cyclin B3-R	TACGCAGTGCCATGAACAGT
cdk1-F	CGTGGTGTATAAGGGTCGCA
cdk1-R	ACGAAATTTCTCTGATCGCGG
rp49-F	CTGCCCACCGGATTCAAG
rp49-R	CGATCTCGCCGCAGTAAAC
